# Detection of gut microbiota and pathogen produced N-acyl homoserine in host circulation and tissues

**DOI:** 10.1038/s41522-021-00224-5

**Published:** 2021-06-28

**Authors:** Jingchuan Xue, Liang Chi, Pengcheng Tu, Yunjia Lai, Chih-Wei Liu, Hongyu Ru, Kun Lu

**Affiliations:** Department of Environmental Sciences and Engineering, University of North Carolina at Chapel Hill, Chapel Hill, NC USA

**Keywords:** Biofilms, Microbiome, Pathogens

## Abstract

Recent studies suggest that quorum-sensing molecules may play a role in gut microbiota-host crosstalk. However, whether microbiota produces quorum-sensing molecules and whether those molecules can trans-kingdom transport to the host are still unknown. Here, we develop a UPLC-MS/MS-based assay to screen the 27 N-acyl homoserine lactones (AHLs) in the gut microbiota and host. Various AHL molecules are exclusively detected in the cecal contents, sera and livers from conventionally-raised mice but cannot be detected in germ-free mice. Pathogen-produced C4-HSL is detected in the cecal contents and sera of *Citrobacter rodentium* (*C. rodentium*)-infected mice, but not found in uninfected controls. Moreover, *C. rodentium* infection significantly increases the level of multiple AHL molecules in sera. Our findings demonstrate that both commensal and pathogenic bacteria, can produce AHLs that can be detected in host bodies, suggesting that quorum-sensing molecules could be a group of signaling molecules in trans-kingdom microbiota-host crosstalk.

## Introduction

Bacterial intercellular communication, known as quorum sensing, plays an important role in a series of bacterial physiological processes, especially in biofilm formation, bacterial cell density, and virulence factor transcription and secretion^1^. Quorum sensing relies on the free-diffused quorum-sensing molecules, also known as autoinducers, which can diffuse to cells of different bacterial species and affect gene expression and behavior. Human gut microbiota contains trillions of microorganisms that play critical roles in food fiber digestion, energy supplement, immune regulation, and so on^2^. However, the gut microbiota is a dynamic and complex system, and its establishment, development, and dysbiosis are still largely unclear. As a key bacteria-bacteria communication approach, quorum sensing could play a role in gut microbiota development. Indeed, multiple quorum-sensing associated genes were detected in the genome of gut bacteria. For example, metagenomics data have shown that many microorganisms in the gut, such as *Hafnia alvei* and *Edwardsiella tarda*, are carrying *LuxI/LuxR* homologs^3^. Our previous study also found multiple quorum-sensing genes and pathways in the metagenome in mouse gut bacteria^4^. However, to our best knowledge, the direct evidence on whether gut microbiota can produce quorum-sensing molecules is still lacking.

In addition, many previous studies revealed that the effects of quorum-sensing molecules are not limited in bacterial cells, and eukaryotes both from plants and mammals could be also affected by these molecules^5^. For example, in plants, bacteria-produced N-acyl homoserine lactones (AHLs), the major quorum-sensing molecules produced by gram-negative bacteria, have been demonstrated to improve systemic resistance of plants by promoting defense gene expression^6,7^. In mammals, the lung pathogen *Pseudomonas aeruginosa* (*P. aeruginosa*) produced AHLs, including 3-OXO-C12-HSL and C4-HSL, can modulate host immune response and affect pathogen clearance^8,9^. Specifically, 3-OXO-C12-HSL can be recognized by receptors of the innate immune system^10^ and selectively impair NF-kB functions in activated human cells which suppress the downstream gene expression^11^. Another study demonstrated that 3-OXO-C12-HSL promoted PPARβ/δ transcriptional activity but inhibited PPARγ transcriptional activity which was associated with 3-OXO-C12-HSL-induced proinflammatory gene expression in murine fibroblasts and human lung epithelial cells^12^. Bacteria-derived AHLs can also promote cell apoptosis by multiple mechanisms^13,14^ and inhibit smooth muscle contraction^15^. Moreover, previous studies have revealed that quorum-sensing autoinducer-2 can regulate interleukin 8 in HCT-8 colon cancer cells and enhance the proliferation of epithelial keratinocytes^16,17^. In mammals, numerous gut microbiota-produced metabolites, such as short-chain fatty acids and secondary bile acids, can enter host bodies to provide important metabolic signals and regulate host gene expression and profoundly modify host health status^2^. These inter-kingdom effects of quorum-sensing molecules raised the possibility that quorum-sensing molecules could be another group of metabolites that participate in the host–microbiota interactions^18,19^. Trans-kingdom transporting to host circulation or specific organs is the prerequisite of quorum-sensing molecules executing their direct and long-range effects on host bodies, however, this type of transportation of gut-sourced quorum-sensing molecules is still not proved yet. For example, *P. aeruginosa* produced AHLs, including C4-HSL and 3OXO-C12-HSL, were only found in the sputum samples but could not be detected in the plasma or urine samples from cystic fibrosis patients with *P. aeruginosa* infection^20,21^.

In this study, we first developed a highly sensitive UPLC-MS/MS-based assay which allows us to detect the trace amount of AHLs in biological samples. By utilizing the germ-free mouse model and *Citrobacter rodentium* (*C. rodentium*)-infected mouse model, we demonstrated that both commensal and pathogenic bacteria in mouse gastrointestinal tracts can produce various AHLs and AHL molecules can be detected in host circulation and liver. This study provides an analytical foundation for the subsequent studies that explore the physiological roles of quorum-sensing molecules in the gut microbiota and host.

## Results

### Performance of the UPLC-MS/MS-based assay for AHL analysis in biological samples

**Fig. 1:**
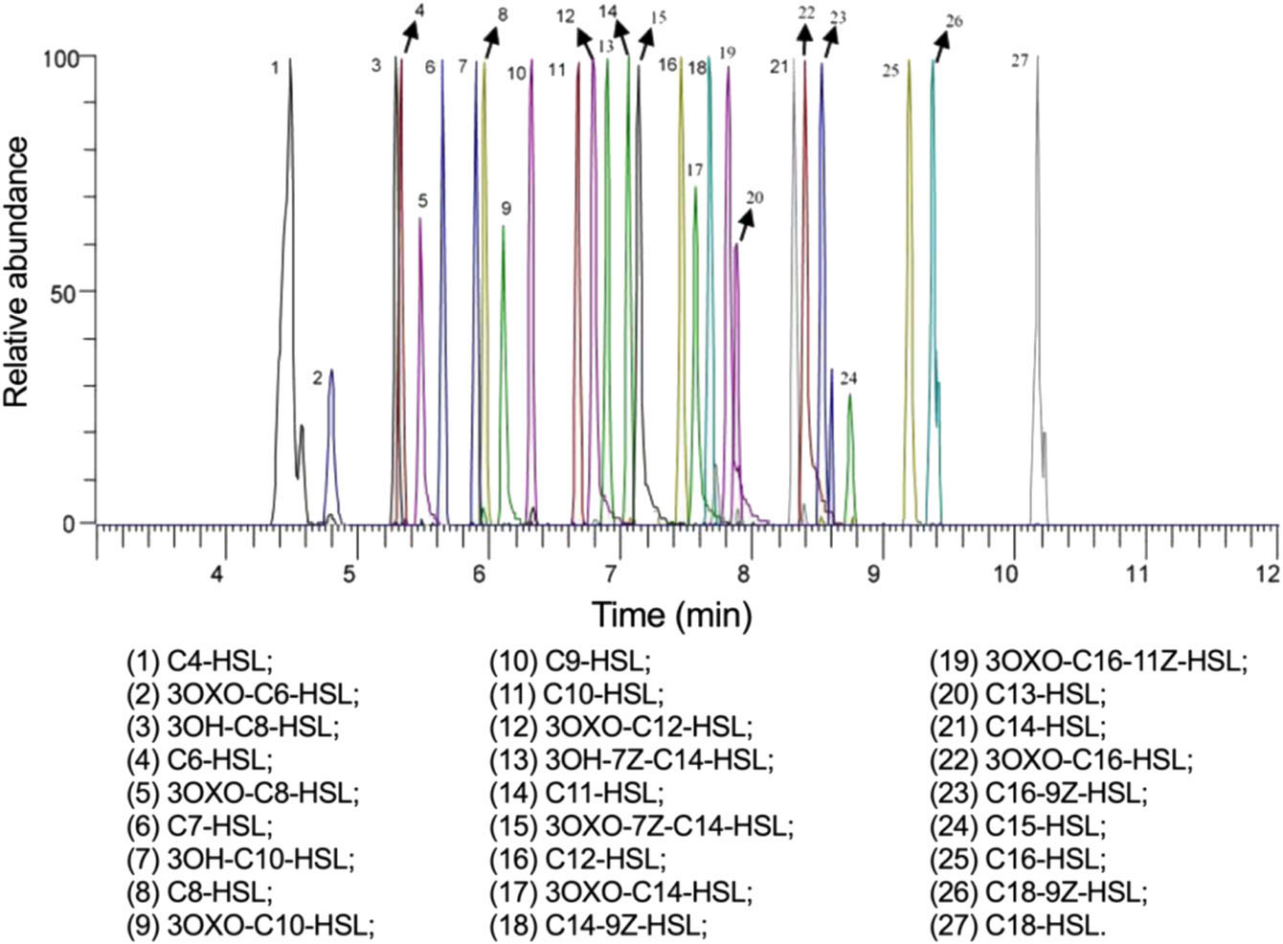
Chromatograms of N-acyl homoserine lactones (AHLs). Typical UPLC-ESI-MS/MS chromatograms of 27 AHLs generated by AHL chemical standards.

Here we developed a highly sensitive UPLC-MS/MS-based assay for the analysis of 27 AHLs, the largest number involved in an analytical protocol for AHLs analysis thus far, in the biological samples, including cecal content, serum, and liver. These AHLs included both saturated and unsaturated AHLs as well as 3OXO- and 3hydroxy-AHLs (Fig. 1; Supplementary Table 1). To ensure the confidence of identification, we used two multiple reaction monitoring (MRM) parameters for each AHL (Supplementary Table 2) as well as the ratios between quantifier and qualifier. Details of the instrumental method optimization, such as mobile phase and column selection, are shown in Supplementary Methods. To favor the detection of trace-level AHLs in the biological samples, the sample pretreatment methods were optimized, and the relevant details are shown in Supplementary Methods. This assay was evaluated with sensitivity, specificity, linearity, matrix effects, assay precision, and accuracy, as shown in Supplementary Tables 3–6. Detailed introductions to these merits of analytical performance were shown in Supplementary Information. Overall, this protocol exhibited superior performance with respect to sensitivity in determining trace-level AHLs in biological samples compared with the currently available methods, with the quantification of limit for the majority of AHLs below nanomolar concentrations.

### Multiple AHL molecules presented in cecal contents, sera, and livers of conventionally-raised mice, but not in samples from germ-free mice

**Fig. 2:**
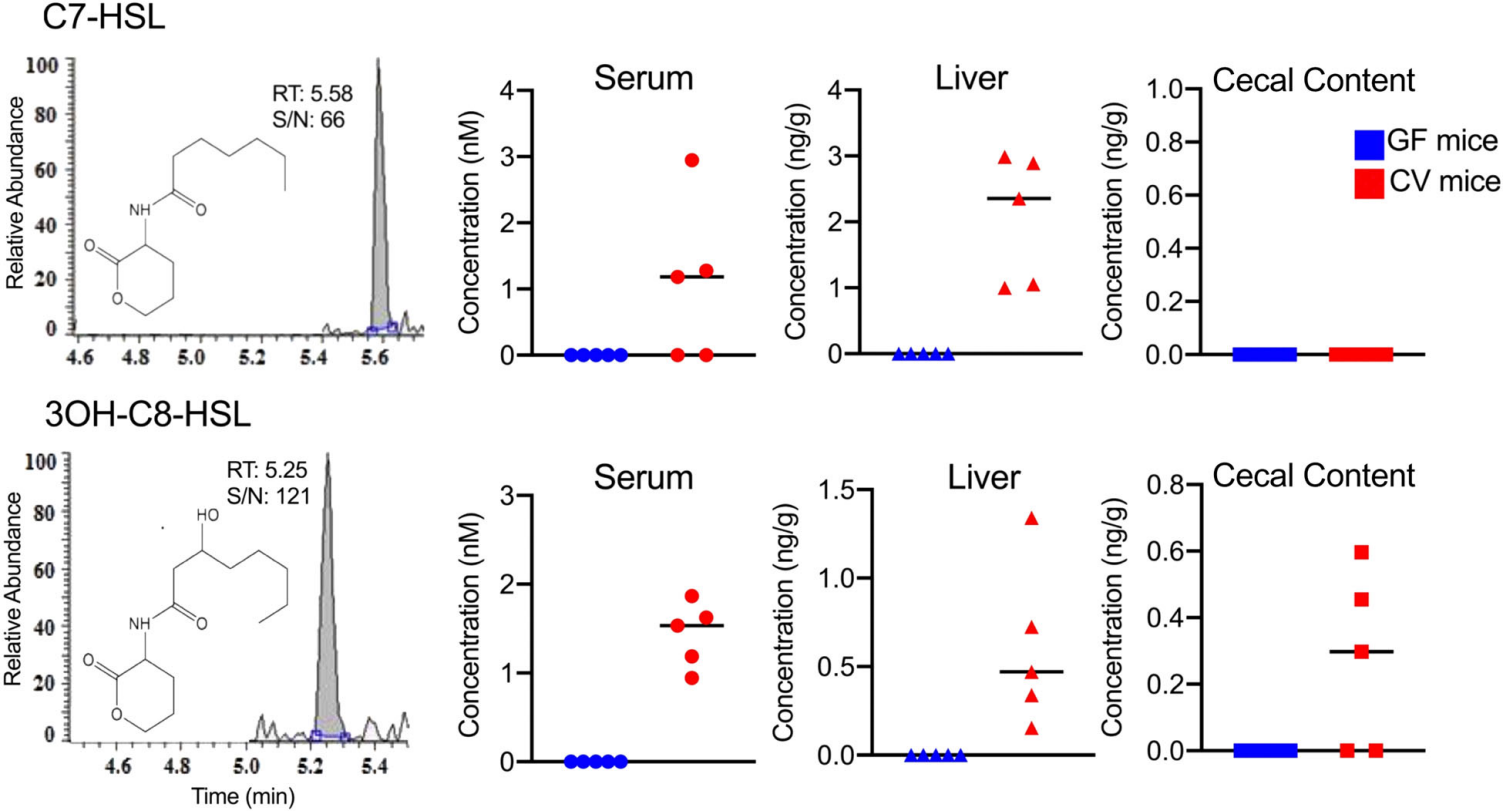
Typical UPLC-ESI-MS/MS chromatograms of short side-chain AHLs (C7-HSL and 3OH-C8-HSL) detected in biological samples and the corresponding concentrations in the biological samples (cecal contents, sera, and livers) of conventional (CV) and germ-free (GF) mice. RT retention time, S/N signal-to-noise ratio.

**Fig. 3:**
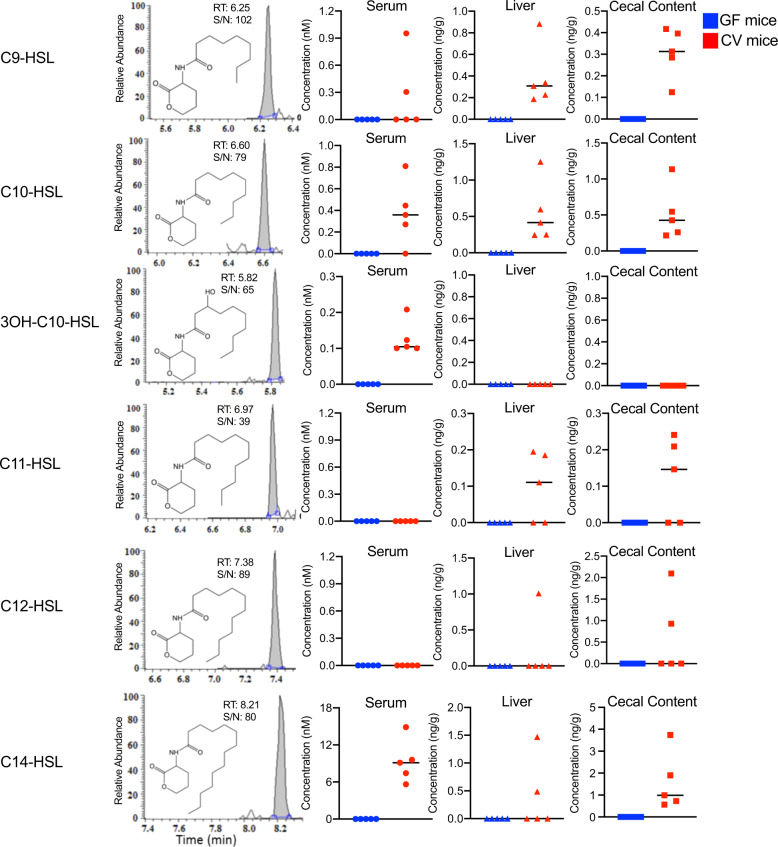
Typical UPLC-ESI-MS/MS chromatograms of long side-chain AHLs (C9-HSL, C10-HSL, 3OH-C10-HSL, C11-HSL, C12-HSL, and C14-HSL) detected in biological samples and the corresponding concentrations in the biological samples (cecal contents, sera, and livers) of conventional (CV) and germ-free (GF) mice. RT retention time, S/N signal-to-noise ratio.

To test the hypothesis that gut microbiota can produce AHLs, we first analyzed the cecal contents in conventionally-raised mice and germ-free mice. As shown in Figs 2 and 3, both short side-chain and long side-chain AHLs were found in the cecal contents from conventionally-raised mice, including 3OH-C8-HSL, C9-HSL, C10-HSL, C11-HSL, C12-HSL, and C14-HSL. Short side-chain AHLs refer to AHLs of <8 carbon atoms on acyl side chain (C_4-8_-HSL) and long side-chain AHLs refer to AHLs with more than 8 carbons on acyl side chain (C_9-18_-HSL)^22^. However, in germ-free animals, none of AHLs could be detected in their cecal contents, indicating that the gut microbes in mice produce multiple types of AHL molecules.

To further investigate whether microbiota-produced quorum-sensing molecules can enter the host, we then analyzed the serum and liver samples from conventionally-raised mice and germ-free mice. We found two short side-chain AHLs, including C7-HSL and 3OH-C8-HSL (Fig. 2), and four long side-chain AHLs, including C9-HSL, C10-HSL, 3OH-C10-HSL, and C14-HSL (Fig. 3), were detected in sera of conventionally-raised mice. In livers, we also found the presence of both short and long side-chain AHLs, including C7-HSL, 3OH-C8-HSL, C9-HSL, C10-HSL, C11-HSL, C12-HSL, and C14-HSL (Figs 2 and 3). However, consistent with the cecal data, none of those AHLs could be detected in germ-free mice, neither in sera nor in livers. Taken together, these results indicate that multiple gut microbiota-derived AHLs can be detected in host circulation and liver.

### C4-AHL produced by the gastrointestinal pathogen *C. rodentium* can be detected in host circulation

**Fig. 4:**
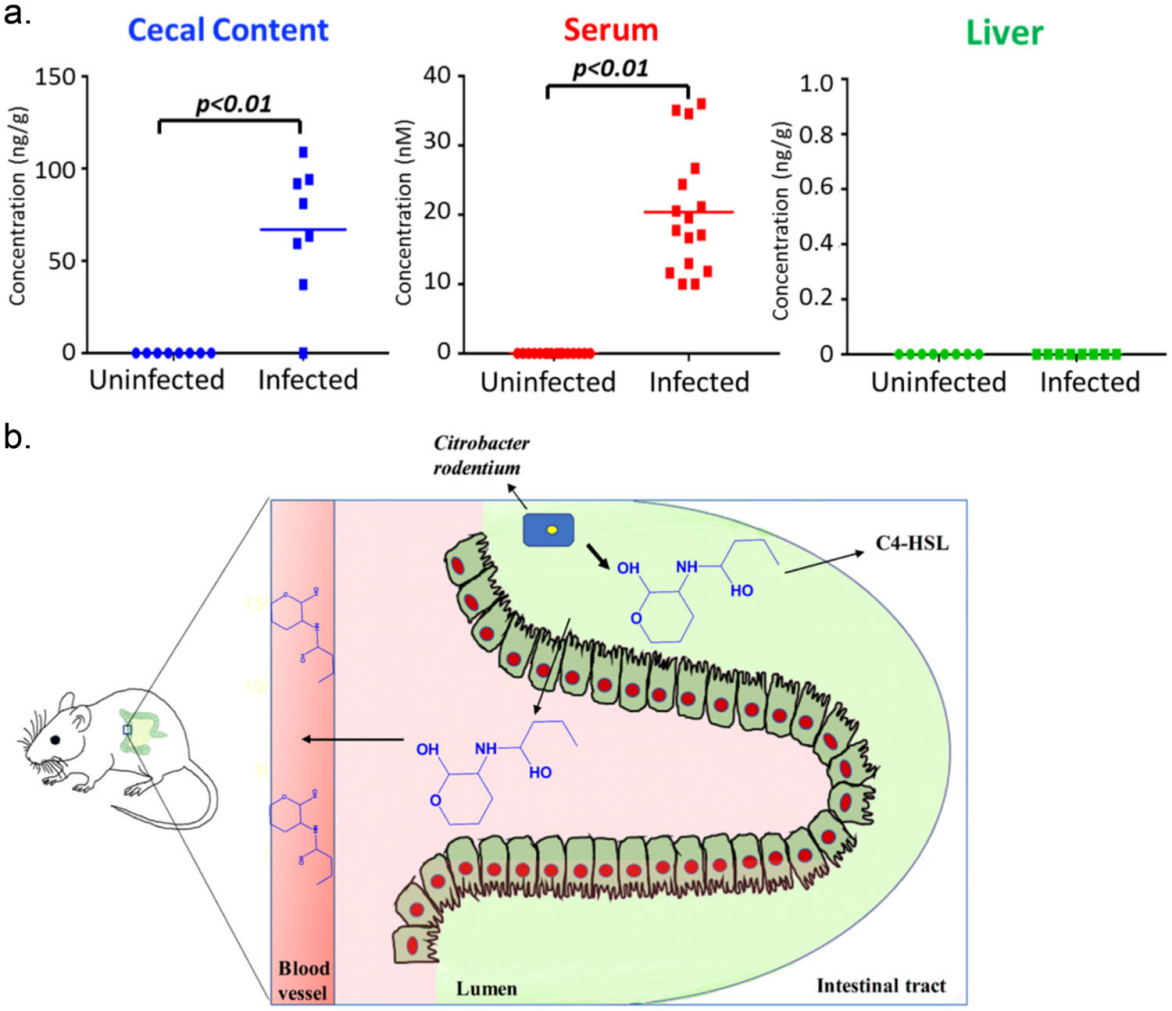
Distribution of C4-HSL in the infected and uninfected mice. **a** Differences of C4-HSL level in the cecal content, serum, and liver between uninfected and infected mice. **b** Schematic representation of in vivo transportation of C4-HSL from *Citrobacter rodentium* to host serum.

*C. rodentium* is a pathogen of mice that restrictively colonized the mouse intestine. A previous study has demonstrated that *C. rodentium* can produce AHL molecules, which plays an important role in the virulence of this pathogen^23^. To investigate the trans-kingdom transportation of gastrointestinal pathogen-produced AHL, we next measured the C4-HSL, one of the major *C. rodentium*-produced AHL molecules suggested by a previous study^23^, in *C. rodentium*-infected mice and uninfected mice. As expected, high-concentrated C4-HSL was detected in the cecal contents of infected mice (70.8 ± 20.0 ng/g), which could not be detected in uninfected mice (Fig. 4a). Correspondingly, infected mice also had a high level of C4-HSL in their sera, but C4-HSL was not detected in any serum samples from uninfected mice (Fig. 4a). C4-HSL was not detected in livers of either infected or uninfected animals (Fig. 4a). These findings demonstrate that C4-HSL, derived from the pathogen *C. rodentium*, can also enter the host circulation, as illustrated in Fig. 4b.

### *C. rodentium* infection perturbed the AHL profiles in the host

**Fig. 5:**
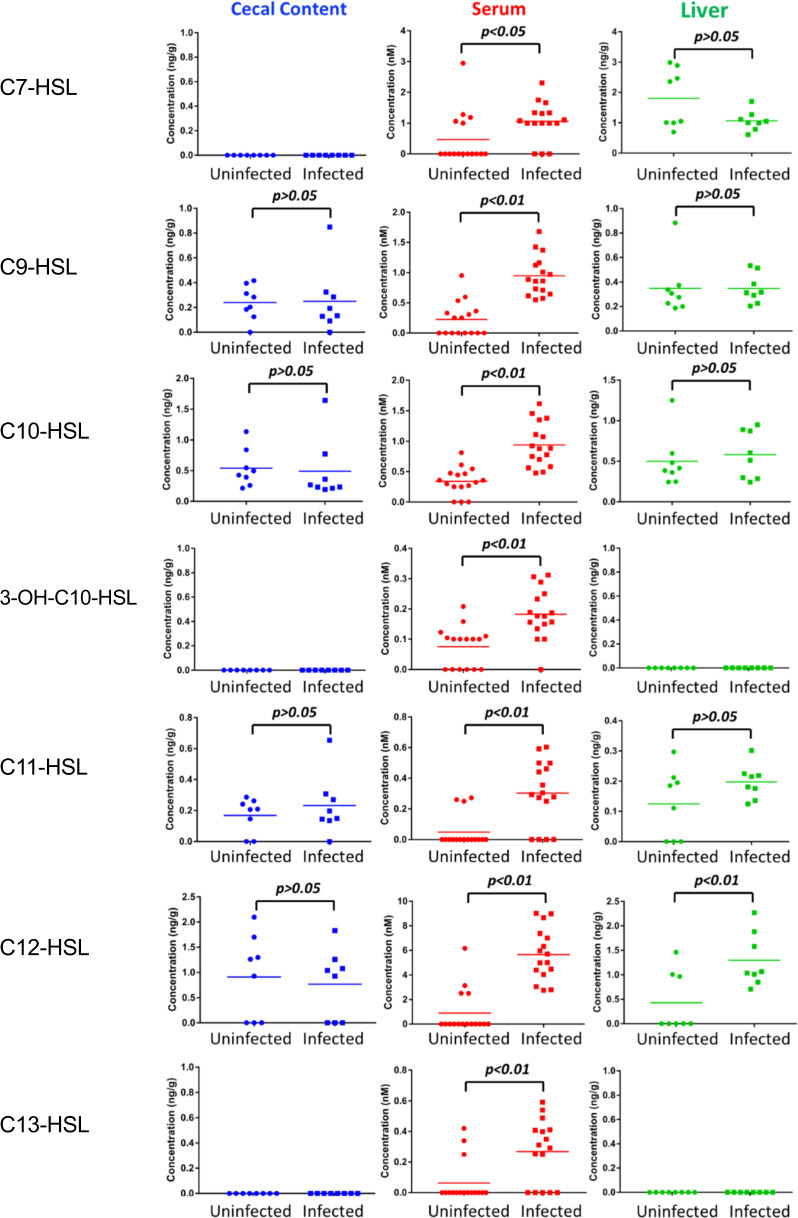
Differences of non-*C. rodentium* produced AHLs level in the biological samples (cecal contents, sera, and livers) between uninfected and infected mice. AHLs in the serum of infected mice were significantly increased compared to non-infected mice.

In addition to C4-HSL, many other AHL molecules were detected both in infected mice and in uninfected mice, including C7-HSL, C9-HSL, C10-HSL, 3OH-C10-HSL, C11-HSL, C12-HSL, C13-HSL, C14-HSL, and 3-oxo-C16-HSL (Fig. 5 and Supplementary Fig. 3). Interestingly, we found most of these detected AHLs were consistently and significantly increased in the sera of *C. rodentium*-infected animals compared with those in uninfected mice (Fig. 5). In livers, C12-HSL in infected mice was significantly higher than in uninfected mice (Fig. 5). However, none of these molecules in cecal contents showed a significant difference between the two groups of mice. Taken together, these results indicate that the profiles of AHLs in the host were disturbed by the *C. rodentium* infection.

## Discussion

Although the role of gut microbiota-associated quorum sensing has been widely discussed^18,24^, there is still no direct evidence indicating that gut microbiota can produce quorum-sensing molecules. Quorum-sensing molecules are notoriously difficult to be detected in the mammalian gut microbiota, potentially due to their structure complexity and low abundance. To our knowledge, only one recent study measured the AHLs in the feces, but not in the body, from patients with inflammatory bowel disease (IBD) based on accurate mass^24^. Here, by developing highly sensitive UPLC-MS/MS-based targeted analytical protocol, we successfully screened 27 AHLs in feces, sera, and mouse tissues. Our study provides a useful method to detect quorum-sensing molecules in biological samples. Moreover, by comparing the conventionally-raised mice and germ-free mice, we first found that mouse gut microbiota produced various AHLs, as demonstrated by the fact that these AHL molecules were only detectable in feces from conventionally-raised mice but not in germ-free mice (Figs 2 and 3). Currently, the specific roles of AHLs in gut microbiota are still unclear, but as the key signaling molecules regulating population density and virulence activation, AHLs might play a regulatory role in the processes of gut microbiota development, maturation, or dysbiosis. This study provides an analytical foundation to further investigate the role of quorum sensing in the gut microbiota.

Another important finding of this study is the trans-kingdom detection of bacteria-derived AHLs in host circulation. By applying germ-free mice and *C. rodentium*-infected mice, we found that AHLs produced by both commensal and pathogenic bacteria can enter into host circulation and to liver (Figs 2–5). Quorum-sensing molecules transporting to eukaryotes have been found in plants. For example, previous studies have detected bacteria-produced AHLs in root and shoot extracts of barley^25,26^. But in mammals, the trans-kingdom transportation of AHLs has not been proved, and the results in our current study clearly demonstrate gut bacteria-produced AHLs can be detected in host bodies.

As mentioned above, the effects of quorum-sensing molecules are not limited in the bacterial community, and many in vitro studies reveal that they also can affect mammalian cells. For example, *P. aeruginosa* produced AHLs can disrupt barrier integrity in human epithelial Caco-2 cells^27,28^ and induce cell apoptosis^13,29,30^. AHLs have also been found with cytotoxicity in multiple cancer cells, such as human pancreatic carcinoma cells and breast carcinoma cells^31,32^. Therefore, the trans-kingdom transportation of microbiota-derived AHLs may have important implications into microbiome–host interaction, because these molecules may not only directly affect the bacterial community and the gut epithelial cells but also have long-range effects in other host tissues. As bacteria-produced functional metabolites analogous to SCFAs and secondary bile acids, AHLs could be a new group of signaling molecules that play a role in host–microbiota interactions. Future studies need to investigate the specific health effects of AHLs and other quorum-sensing molecules in the host.

Our study also found that the abundance of AHLs in host bodies was dramatically perturbed under the context of *C. rodentium* infection (Figs 4 and 5). In addition to the dramatic increase of *C. rodentium* produced C4-AHL (Fig. 4), many other AHL molecules were also significantly increased in mouse sera (Fig. 5). Since no evidence is showing that *C. rodentium* can produce these molecules thus far, these AHLs may be originated from other gut bacteria. The changed AHL profiles could be caused by the alterations in gut microbial composition in the context of *C. rodentium* infection, but further work needs to be done to test this hypothesis.

The significant changes of AHLs in infected mice have two important implications. First, as gut microbiota generated signaling molecules are likely produced to sense community density, AHL abundance and composition could serve as biomarkers indicating the changes of gut microbiota. Our previous study found malathion exposure significantly disturbed the development of mouse gut microbiota characterized by the wide alterations in abundance of quorum sensing related genes^33^. A recent study also associated the gut microbiota perturbation with the fecal AHL changes in IBD patients^24^. Second, considering the biological effects of AHLs on mammalian cells as demonstrated by many in vitro assays, changed AHL profiles could have some health effects in host bodies, in which the AHL abundance/composition may serve as important metabolic signaling in host–microbiota interaction. Multiple studies have demonstrated that AHLs are not only quorum-sensing molecules for intra- and inter-species communication among bacteria, but also have biological effects on mammals. For example, AHLs can disrupt intestinal epithelial barrier by decreasing the tight junction proteins^34^. Previous studies also found many other health effects of AHLs, such as mobilizing intracellular calcium^35^, induce inflammation by activating phagocytosis in human phagocytes^36^ inhibiting mammalian cell proliferation, and causing cell death in a variety of cell types^31,32,37^. Therefore, changing AHLs in the host could be a potential mechanism underlying host health problems induced by diverse factors^4,24,38^. For example, in this study, we found both C4-HSL and C12-HSL were significantly increased in *C. rodentium*-infected animals (Figs 4 and 5). A previous study demonstrated that exposing C4-HSL to mouse and human immune cells could suppress T-cell proliferation and decreased levels of cytokines (IFN-γ and IL-4)^39^. The effects of C4-HSL on the host immune system might exacerbate the pathogen infection. Likewise, C12-HSL, one of the most abundant AHL in mouse sera we detected, has been found that can eliminate key defense cells by triggering apoptosis^40^, which might also aggravate the effects of pathogen infection. Current knowledge about the specific health effects of gut bacteria-derived AHLs are still very limited but future studies in this area will deepen our understanding of host–microbiota interactions.

Interestingly, there was no difference for these AHL molecules in cecal contents between uninfected and infected mice (Fig. 5). In particularly, several AHLs were not detectable in fecal samples of either uninfected or infected mice, however, their concentrations clearly increased in the serum of infected animals. Several reasons may lead to such an observation. For example, it could be caused by the rapid AHL degradation in gut epithelia^41–44^. Indeed, a previous study proved that human and mouse gastrointestinal tract expressed paraoxonases, including PON1, PON2, and PON3, and importantly, PON1 and PON2 were secreted to the environment^44^. Both PON1 and PON2 have lactonase activity which can hydrolyze AHLs^45^. Likewise, intestinal cells might actively absorb and transport the bacteria-derived AHLs which thus eliminate AHL abundance difference between uninfected and infected mice. Moreover, the increase of AHLs in infected animal may arise from elevated gut permeability. Regardless, it should be noted that the status of AHLs in feces may not correctly reflect the AHLs in host circulation, as we observed in the *C. rodentium* mouse model.

Another interesting finding is that the effects of infection on AHL profiles in sera were very different from those in livers. First, the wide increases of AHLs in sera from infected mice were not observed in livers (Fig. 5); moreover, even some AHLs had high concentrations in the serum, such as C4-HSL, they could not be detected in liver tissues (Fig. 4). This suggests that the transportation of some AHLs from serum to liver may be selective but not by passive diffusion. A previous study demonstrated that 3-OXO-C12-HSL could be actively expelled from several types of mammalian cells by ABC transporter ABCA1, including A549 human lung epithelial cells, primary mouse embryonic fibroblasts (MEFs), and NIH3T3 mouse fibroblasts^46^. It is still unclear whether hepatocytes can also actively transport AHLs or not. Future studies need to address the underlying mechanism and the associated biological effects of the tissue-specific AHL profiles.

In summary, this study demonstrates that gut microbiota can produce various AHLs that can further transport into the host. Deciphering the role of quorum sensing in gut microbiota and host–microbiota interaction will deepen our understanding about the microbiome/pathogen-host crosstalk. AHLs may serve as promising biomarkers for microbiome changes or bacterial infection. Moreover, it may provide a new efficient approach to manipulate gut microbiota. For example, a previous study successfully transferred signals from one gut bacterial species to another one by AHLs^47^. Another study went further to use engineered *Escherichia coli* to alter the composition of the antibiotic-treated gut microbiota by increasing intestinal autoinducer-2^48^. Quorum-sensing system has been considered as a promising therapeutic target for bacterial diseases^49^.

## Methods

### Chemicals and reagents

Analytical standards of N-butanoyl-L-homoserine lactone (C4-HSL), N-hexanoyl-L-homoserine lactone (C6-HSL), N-heptanoyl-L-homoserine lactone (C7-HSL), N-(*β*-ketocaproyl)-L-homoserine lactone (3-OXO-C6(L)-HSL), N-octanoyl-L-homoserine lactone (C8-HSL), N-(3-oxooctanoyl)-L-homoserine lactone (3-OXO-C8-HSL), N-nonanoyl-L-homoserine lactone (C9-HSL), N-(3-hydroxyoctanoyl)-L-homoserine lactone (3-OH-C8-HSL), N-decanoyl-L-homoserine lactone (C10-HSL), N-undecanoyl-L-homoserine lactone (C11-HSL), N-3-hydroxydecanoyl-L-homoserine lactone (3-OH-C10-HSL), N-dodecanoyl-L-homoserine lactone (C12-HSL), N-tridecanoyl-L-homoserine lactone (C13-HSL), N-*cis*-tetradec-9Z-enoyl-L-homoserine lactone (C14-9Z-HSL), N-tetradecanoyl-L-homoserine lactone (C14-HSL), N-3-oxo-tetradec-7(Z)-enoyl-L-homoserine lactone (3-OXO-7Z-C14-HSL), N-(3-hydroxy-7-*cis* tetradecenoyl)-L-homoserine lactone (3-OH-7Z-C14-HSL), N-pentadecanoyl-L-homoserine lactone (C15-HSL), N-3-oxo-tetradecanoyl-L-homoserine lactone (3-OXO-C14-HSL), N-*cis*-hexadec-9Z-enoyl-L-homoserine lactone (C16-9Z-HSL), N-hexadecanoyl-L-homoserine lactone (C16-HSL), N-3-oxo-hexadec-11(Z)-enoyl-L-homoserine lactone (3-OXO-C16-11Z-HSL), N-3-oxo-hexadecanoyl-L-homoserine lactone (3-OXO-C16-HSL), N-*cis*-octadec-9Z-enoyl-L-homoserine lactone (C18-9Z-HSL), and N-octadecanoyl-L-homoserine lactone (C18-HSL) were purchased from Cayman Chemical (Ann Arbor, MI, USA). Analytical standards of N-(3-oxodecanoyl)-L-homoserine lactone (3-OXO-C10-HSL) and N-(3-oxododecanoyl)-L-homoserine lactone (3-OXO-C12-HSL) were obtained from Sigma-Aldrich Co. (St. Louis, MO, USA). Internal standard (IS) of C6-HSL (d_3_-C6-HSL) was from Cayman Chemical. Stock solutions (1–100 mM) of the 27 AHLs were individually prepared from their authentic compounds in DMSO/chloroform/acetonitrile based on their solubility. D_3_-C6-HSL was prepared at 20 mM in DMSO and used as the IS solution for all the AHLs measured. All the stock solutions were stored at −80 °C and further diluted with methanol to prepare working solutions. Final calibration solutions were prepared in 50% methanol with a concentration range of 0.01–100 nM. LC-MS grade acetonitrile, methanol, DMSO, water, formic acid, and other solvents were obtained from Sigma-Aldrich.

### Sample collection

#### Mouse sample collection

A total of 5 specific-pathogen-free (SPF) C57BL/6 mice (~8-week-old) were purchased from Jackson Laboratories and housed in the animal facility of the University of North Carolina at Chapel Hill for 1 week to acclimate with standard pelleted rodent diet and tap water ad libitum provided. Then the mice were housed under the environmental conditions of 22 °C, 40–70% humidity, and a 12:12 h light/dark cycle and were provided water ad libitum throughout the experiment period. Regular monitoring for health conditions was twice a week. After 7 weeks, serum, urine, liver, cecal content, and feces samples from individual mouse were collected and kept at −80 °C immediately for further analysis. Germ-free mice (~8-week-old) were maintained in UNC germ-free animal facility and samples of germ-free mice were collected and stored as SPF mice. The animal protocol was approved by the University of North Carolina at Chapel Hill Institutional Animal Care and Use Committee.

### Pathogen infection

A total of 20 *Citrobacter*-free C57BL/6 (~8-week-old) mice, from Jackson Laboratories (Bar Harbor, ME), were provided with pelleted rodent diet (ProLab 3000, Purina Mills, St. Louis, MO) and filtered water ad libitum. The animals were maintained in AAALAC-accredited facilities in microisolator caging under standard environmental conditions. All experiments were approved by the Massachusetts Institute of Technology Committee on Animal Care. In the beginning of infection treatment, mice were randomly assigned to either control or treatment group (*Citrobacter rodentium* dose), with each grouping comprised of 10 mice. Mice were dosed at 9–10 weeks of age with 2 × 10^7^ *Citrobacter rodentium* three times on alternate days by oral gavage. The colonization were monitored by measuring colony-forming units (CFU) in stool samples, which reached ~1.1 × 10^9^ CFU/g feces at 7 days post infection. Serum and cecal content samples were collected during necropsy at the end of the study.

### Sample preparation methods development

#### Serum

Four different sample preparation approaches were investigated for the extraction of AHLs in serum sample: protein precipitation (PPT), protein precipitation followed by reversed-phase solid phase extraction (PPT-SPE), normal phase solid phase extraction (NP-SPE), and phospholipid-depletion solid phase extraction (PD-SPE). The details of the PPT, PPT-SPE, NP-SPE, and PD-SPE methods are described as following:

*PPT*. Each 20-µL aliquot of the serum or urine sample was mixed with 20 µL of 100 nM *d*_3_-C6-HSL solution (in methanol) and 160 µL of cold methanol (0.1% FA). After vortex mixing for 15 s, the tubes were placed at −20 °C for 30 min, followed by centrifugation at 21,130 × *g* at room temperature for 10 min. Then the supernatants were collected and speed vacuum dried. After resuspension with 100 µL 50% MeOH (0.1% FA), the solution was centrifuged at 21,130 × *g* at room temperature for 10 min before the aliquot was taken for instrumental injection.

*PPT-SPE*. Each 20-µL aliquot of the serum sample was mixed with 20 µL of 100 nM *d*_3_-C6-HSL solution (in methanol) and 160 µL of cold methanol (0.1% FA). After vortex mixing for 15 s, the tubes were placed at −20 °C for 30 min, followed by centrifugation at 21,130 × *g* at room temperature for 10 min. Then the supernatants were collected and speed vacuum dried. 100 µL of methanol was added to the tube and vortex mixed for 30 s, followed by the addition of 1.5 mL of water (0.1% FA). Then the mixture was loaded onto an ISOLUTE C18 cartridge (100 mg/1 mL). Before sample loading, the cartridge was washed with 2 mL of methanol and conditioned with 2 mL of acidified water (0.1% FA). Followed the sample loading, the cartridge was washed with 2 mL of acidified water (0.1% FA). After vacuum drying, the analytes were eluted with 750 × 2 µL of acidified methanol (0.1% FA). The elutes were speed vacuum dried at 8 °C, then resuspended in 100 µL of 50% methanol (0.1% FA).

*NP-SPE*. Each 20-µL aliquot of the serum or urine sample underwent the protein precipitation procedure as described in *PPT* section. After the supernatants were speed vacuum dried, 100 µL of dichloromethane was added to the tube and vortex mixed for 30 s, followed by the addition of 1.5 mL of hexane (0.1% FA). The mixture was loaded onto a Strata NH2 (55 µm, 70 Å) cartridge (100 mg/1 mL). Before sample loading, the cartridge was washed with 2 mL of methanol and conditioned with 2 mL of hexane (0.1% FA). Followed the sample loading, the cartridge was washed with 2 mL of hexane (0.1% FA). After vacuum drying, the analytes were eluted with 750 × 2 µL of acidified methanol (0.1% FA). The elutes were speed vacuum dried at 8 °C, then resuspended in 100 µL of 50% methanol (0.1% FA).

*PD-SPE*. Each 20-µL aliquot of the serum or urine sample was mixed with 20 µL of 100 nM *d*_3_-C6-HSL, 100 µL of water (0.1% FA), and 360 µL of acetonitrile (0.1% FA). After vortex mixed for 30 s, the tubes were centrifuged at 21,130 × *g* at room temperature for 10 min. Then the supernatant was collected and loaded onto a HybridSPE Phospholipid Ultra SPE tube (30 mg/1 mL), which was washed with 2 mL of methanol and conditioned with 70% aqueous acetonitrile containing 0.1% FA prior to use. After sample loading, the flow-through fractions were collected into 2 mL Eppendorf tubes. Then the SPE tube was washed with 1.2 mL of 70% aqueous acetonitrile (0.1% FA). The flow-through fractions were also collected and combined before speed vacuum dried. The residues were reconstituted in 100 µL of 50% methanol (0.1% FA).

#### Liver and cecal content

Two different sample preparation protocols were evaluated to optimize the extraction efficiency of AHLs from liver and cecal content samples: extraction with methanol and DCM followed by RP-SPE and NP-SPE. Detailed procedure for RP-SPE and NP-SPE are described as following:

*RP-SPE*. Approximately 20 mg of biological tissue (liver and cecal content) sample was weighed into a 1.5 mL Eppendorf tube and extracted with 800 µL of methanol and dichloromethane:methanol (1:1, v:v) in order. For each extraction, a tissuelyser was operated at 50 Hz for 15 min, followed by centrifugation at 21,130 × *g* for 10 min. Then both supernatants were combined and centrifuged again before collecting the supernatant for speed vacuum dry. One hundred microliters of methanol were used for reconstitute, then diluted to 1.5 mL with water (0.1% FA) after vortex mixing for 30 s. The mixture was passed through an ISOLUTE C18 cartridge (100 mg/1 mL), which was washed with 2 mL of methanol and conditioned with 2 mL of water (0.1% FA) prior to analysis. Followed the sample loading, the cartridge was washed with 2 mL of water (0.1% FA). After vacuum drying, the analytes were eluted with 700 × 2 µL of methanol (0.1% FA). The elutes were speed vacuum dried at 8 °C, then resuspended in 100 µL of 50% methanol (0.1% FA) for instrumental injection.

*NP-SPE*. Approximately 20 mg of biological tissue (liver and cecum) or fecal sample was weighed into a 1.5 mL Eppendorf tube and extracted with 800 µL of methanol and DCM:methanol (1:1, v:v) in order. For each extraction, a tissuelyser was operated at 50 Hz for 15 min, followed by centrifugation at 21,130 × *g* for 10 min. Then both supernatants were combined and centrifuged again before collecting the supernatant for speed vacuum dry. One hundred microliters of DCM were used for reconstitute, then diluted to 1.5 mL with hexane (0.1% FA) after vortex mixing for 30 s. The mixture was passed through a Strata NH2 (55 µm, 70 Å) cartridge (100 mg/1 mL), which was washed with 2 mL of methanol and conditioned with 2 mL of hexane (0.1% FA) prior to analysis. Followed the sample loading, the cartridge was washed with 2 mL of water (0.1% FA). After vacuum drying, the analytes were eluted with 700 × 2 µL of methanol (0.1% FA). The elutes were speed vacuum dried at 8 °C, then resuspended in 100 µL of 50% methanol (0.1% FA) for instrumental injection.

### Optimization of sample preparation method

PPT is the most commonly used analytical protocol for extracting chemicals from biofluids. It has been reported that methylene chloride can extract 3-OXO- and 3-hydroxy-HSLs more efficiently^50^. However, this study didn’t observe significant differences in both the types and peak areas of AHLs extracted by methanol and methylene chloride. Passage through C18 SPE cartridge not only helps detect more types of AHLs, but also increase the corresponding peak areas. This clearly indicates that matrix effect is suppressing the intensity of AHLs in biofluids. Besides the RP-SPE cartridge, normal phase (NP) SPE cartridge was tested because NP-SPE was reported to be effective in separating AHLs from contaminants due to the polar nature of the homoserine lactone ring shared by AHLs^50^; HybridSPE column was also tested because it has been reported that HybridSPE column can successfully remove the phospholipids in serum samples, which helps reduce matrix effects in LC-ESI-MS analysis^51^. Supplementary Fig. 1A shows bar graphs of measured peak areas plus the standard deviations (SD) of the AHLs detected by each method. To test the extraction efficiency of those AHLs not present in the test samples, matrix spiked samples were used and bar graphs of the area ratios plus the SD of six representative AHLs are shown in Supplementary Fig. 1B. Overall, PPT-SPE demonstrated good extraction efficiency and lower SDs compared with other analytical approaches. PD-SPE can obtain higher peak areas (or ratios) for some AHLs but this method also generates higher SDs. Therefore, in this study, PPT-SPE was employed to improve the capability in detecting more types of AHLs in serum samples.

For the sample preparation of biological tissue samples, both RE-SPE and NP-SPE were used to investigate the extraction efficiency of AHLs. Similar to the strategy employed for serum samples, both original liver samples and liver samples spiked with AHLs standards were used, as shown in Supplementary Fig. 2A, B, respectively. NP cartridge shows better recovery for C4-HSL, while RP cartridge exhibits equal or better recoveries for all other AHLs, especially those with long side-chain. RP-SPE extraction protocol demonstrated equal or better recoveries for the majority of AHLs measured in this study, compared with NP-SPE; thus, RP-SPE was selected as the sample preparation protocol for liver and cecal content samples in this study.

### UPLC-MS/MS methods development

#### Instrumental method

A Vanquish UHPLC system (Thermo Scientific, Waltham, MA, USA) coupled to a TSQ Quantis triple quadrupole mass spectrometer (Thermo Scientific) via an electrospray ionization (ESI) source operated in positive ion mode. An ACQUITY UPLC HSS T3 (2.1 mm × 100 mm, 1.8 µm) column (Waters Inc., Milford, MA, USA) was used for the chromatographic separation of AHLs, with mobile phase A of 0.1% FA in water and mobile phase B of 0.1% FA in acetonitrile, respectively. The AHLs were eluted at a flow rate of 200 µL/min by gradient elution of mobile phase starting at 5% B, increased to 10% B in 1 min; then increased to 75% B in 3 min and further increased to 99% B at 6 min, then held for 6.5 min; then decreased to 5% B at 13 min, and held for 3.5 min, for a total run of 16.5 min. The injection volume was 10 µL and the column was maintained at 50 °C. The source parameters were set as follows: spray voltage, 4500 V; sheath gas, 40 arbitrary unit; aux gas, 10 arbitrary unit; sweep gas, 6 arbitrary unit; ion transfer tube temperature, 350 °C; vaporizer temperature, 450 °C.

#### Optimization of instrumental method

Positive ionization mode was selected since AHLs showed a greater sensitivity when positively charged. Besides the protonated [M + H]^+^ species, this family of compounds also tends to form adducts and clusters such as [M + Na]^+^, [M + CH_3_OH + H]^+^, [M + K]^+^, and [M + Acetonitrile+Na]^+^, in positive ionization mode. However, there were no stable and sensitive fragments formed in the collision cell for these adduct ions of AHLs. Protonated [M + H]^+^ species of AHLs generate two intense and characteristic fragmentations in the collision cell in triple quadrupole mass spectrometry: an ion corresponding to the lactone moiety at *m*/*z* 102 and an ion derived from the acyl chain moiety [M + H-101]^+^. For the AHLs measured in this study, the two fragments provide better sensitivity than any other transitions: the fragment ion with higher intensity was used as the quantifier and the one with lower intensity was qualifier (Supplementary Table 2). The ratio between quantifier and qualifier was also used for the identification of AHLs in biological samples. Optimization of the MRM transitions for 27 AHLs was conducted by direct injection for the individual standard solution prepared in 50% methanol (0.1% formic acid (FA)). Their optimized MRM transitions are summarized in Supplementary Table 2. The ESI-MS parameters such as voltage, drying gas, and vaporizer temperature are optimized based on the flow rate of mobile phase and the acquired peak areas of AHLs after injecting the standard mixture.

To achieve better sensitivity for the 27 AHLs involved in the protocol development, mobile phases with different compositions were tested and measured peak areas of AHLs showed that mobile phases of 0.1% FA in water (A) and 0.1% FA in acetonitrile (B) were the optimal mobile phases. Different resuspension solvents were also tested to optimize the sensitivity of AHLs. AHLs with short side-chain showed better sensitivity in resuspension solvent with more water, however, AHLs with long side-chain showed better sensitivity in resuspension solvent with more organic solvent. In general, AHLs showed higher intensity in methanol compared with acetonitrile, especially for those long side-chain AHLs. Thus, 50% methanol containing 0.1% FA was chosen as the resuspension solvent for AHLs measurement. Addition of FA aims to reduce the amount of lactone hydrolysis during storage^52^. Although there were no structural isomers among the 27 AHLs measured, exact masses of 3-OXO-C_n-1_-HSL and C_n_-HSL could not be distinguished by low-resolution mass spectrometry, thus the two chemicals share the same MRM transitions. 3-OXO-C14-HSL, C15-HSL, and 3-OH-7Z-C14-HSL also have the same MRM transition. Due to the inherent lipophilic nature of AHLs, several reversed-phase (RP) HPLC columns were tested. The HSS T3 10 cm long column gave the best separation with the mobile phases and resuspension solvent mentioned above.

#### Sensitivity and linearity

In the present study, *d*3-C6-HSL, an isotope-labeled internal standard for C6-HSL, was used as the common internal standard for all the AHLs measured to correct the analytical variations from sample preparation. Ten-point calibration curves with concentrations ranging from 0.01 to 100 nM were established for each analyte using the ratio of peak areas of AHLs and the internal standard (*d*3-C6-HSL) as a function of the nominal concentrations of AHLs in solvent. The regression coefficients of all calibration curves were >0.99, as summarized in Supplementary Table 2. The signal-to-noise (S/N) ratios measured from a series of diluted standard solutions of the 27 AHLs were used to estimate the lower limit of detection (LLOD) for each AHL, defined as the lowest concentration that would yield at least three times the S/N ratio (Supplementary Table 3). The lower limits of quantification (LLOQs) of AHLs in serum and liver were determined as the lowest concentration in the matrix-matched calibration curve with 10 times the S/N ratio (Supplementary Table 4). LLOQs of AHLs in cecum are considered as the same as those in liver.

#### Matrix effects

The matrix effect was expressed as the ratio of the mean peak area of the analytes spiked in the final sample extract prior to instrumental analysis to the mean peak area of the same standard spiked in the solvent, multiplied by 100^53^. A value of >100% indicates ionization enhancement, and a value of <100% indicates ionization suppression^53^. Matrix effects of AHLs in serum and liver are summarized in Supplementary Table 5. Cecum is considered to have the same matrix effects as liver for AHLs. Ionization suppression for all the AHLs measured was observed in liver samples, while not in serum.

#### Assay precision and accuracy

The assay precision was determined as the intra-day and inter-day relative standard deviations (RSDs). Both serum and liver samples were spiked at two different fortification levels of the target analytes: low (MS-L) and high (MS-H). For intra-day precision, six (*n* = 6) replicate analyses were performed at each level within 1 day; for inter-day precision, six (*n* = 6) replicate analyses were performed at each level on 3 continuous days. Supplementary Table 6 lists the intra-day and inter-day % RSDs for 27 AHLs at both concentration levels in serum and liver. The intra-day RSDs were determined to be from 4.33% to 29.7% and from 2.58% to 26.4%, respectively, in serum and liver samples. The inter-day RSDs were from 1.46% to 28.8% and from 2.25% to 29.4%, respectively, in serum and liver samples. These measured RSDs indicated good quantitation precision for all the AHLs measured in this study.

Assay accuracy was evaluated using recovery tests conducted on 3 consecutive days in ten replicate analyses in serum and liver samples. Percent recoveries (%) were determined as the ratios between measured concentrations in matrix spiked samples (standards were spiked into the samples prior to extraction) divided by those measured in matrix-matched samples (standards were spiked into the final extracts of samples). The mean relative recoveries of AHLs at both low and high concentrations were summarized in Supplementary Table 6. The majority of AHLs demonstrated acceptable recoveries (70–130%) relative to *d*3-C6-HSL in both serum and liver samples, indicating our analytical protocol offered accurate quantitation for the AHLs measured in this study.

### Data analysis

#### Quality assurance/quality control (QA/QC)

For the quantification of AHLs in the samples, the isotope dilution method was employed based on the response of *d*_3_-C6-HSL. A ten-point calibration curve was prepared for each analyte with concentrations ranging from 0.01 to 100 nM and regression coefficient (*r*) of each calibration curve is ≥0.99. Pure solvent (methanol) and a mid-point calibration standard was injected every 6 samples as a check for carry over across samples, for the drift in instrumental sensitivity, and for the confirmation of retention time of AHLs. Although AHLs are endogenous toxicants produced by gut bacteria, procedural blanks were also prepared and injected. As expected, no AHLs were found in the blanks.

The lower limit of detection (LLOD) was determined with serial dilution of the AHL mixture and the lower limit of quantification (LLOQ) was defined with serial dilution of the AHLs mixture spiked into the relevant matrix. Matrix effects were calculated by comparing the instrumental responses between solvent-based and matrix-matched calibration standards. The accuracy and precision of the method were examined by the analysis of AHLs-fortified samples. Matrix spike samples were prepared at two different concentrations of AHLs: low (serum: C4-HSL, 2 nM; others: 1 nM. liver: C4-HSL, 8 nM; others, 4 nM) and high (serum: C4-HSL, 10 nM; others: 5 nM. liver: C4-HSL, 30 nM; others, 15 nM).

### Statistical analysis

Thermo Scientific XCalibur was used to acquire MS/MS data. Statistics software package R v.3.1.0 and Microsoft Excel 2007 were used for statistical analysis. For the calculation of arithmetic mean (mean) and standard deviation (SD) of left-censored data, robust regression-on-order statistics (ROS) was employed. GraphPad Prism 7.04 was used for the preparation of the scatter plots of the AHLs levels in the samples. To test the difference of the levels of AHLs between two groups, a Student’s *t*-test (when data follow a log-normal distribution) or a Mann–Whitney *U* test (when data does not follow a log-normal distribution) was used. A Shapiro–Wilk test and Quantile–Quantile (Q–Q) plot were used to investigate the normality assumption of the data. The levels of AHLs are presented at a unit of nM in serum and at a unit of ng/g in liver and cecal content. The statistical significance level was set at *p* < 0.05.

### Reporting summary

Further information on research design is available in the Nature Research Reporting Summary linked to this article.

## Supplementary information

Reporting Summary

Supplementary Information

## Data Availability

All data generated in method development are provided in Support Information. The source data underlying Figs 1–5 and all other data are available from the corresponding author on reasonable request.
