# Supplementary material for: Detection of gut microbiota and pathogen produced N-acyl homoserine in host circulation and tissues
Source: NPJ Biofilms Microbiomes. 2021 Jun 28;7:53. doi: 10.1038/s41522-021-00224-5 (PMC8239043; doi:10.1038/s41522-021-00224-5)
Supplement: Supplementary file 1 — Reporting Summary [file 41522_2021_224_MOESM1_ESM.pdf]

nature research

Corresponding author(s): Kun Lu
Last updated by author(s): Apr 16, 2021

Reporting Summary

Nature Research wishes to improve the reproducibility of the work that we publish. This form provides structure for consistency and transparency in reporting. For further information on Nature Research policies, see our Editorial Policies and the Editorial Policy Checklist.

Statistics

For all statistical analyses, confirm that the following items are present in the figure legend, table legend, main text, or Methods section.

n/a	Confirmed	
☐	☒	The exact sample size (*n*) for each experimental group/condition, given as a discrete number and unit of measurement
☒	☐	A statement on whether measurements were taken from distinct samples or whether the same sample was measured repeatedly
☐	☒	The statistical test(s) used AND whether they are one- or two-sided<br>*Only common tests should be described solely by name; describe more complex techniques in the Methods section.*
☒	☐	A description of all covariates tested
☒	☐	A description of any assumptions or corrections, such as tests of normality and adjustment for multiple comparisons
☐	☒	A full description of the statistical parameters including central tendency (e.g. means) or other basic estimates (e.g. regression coefficient) AND variation (e.g. standard deviation) or associated estimates of uncertainty (e.g. confidence intervals)
☒	☐	For null hypothesis testing, the test statistic (e.g. *F*, *t*, *r*) with confidence intervals, effect sizes, degrees of freedom and *P* value noted<br>*Give P values as exact values whenever suitable.*
☒	☐	For Bayesian analysis, information on the choice of priors and Markov chain Monte Carlo settings
☒	☐	For hierarchical and complex designs, identification of the appropriate level for tests and full reporting of outcomes
☒	☐	Estimates of effect sizes (e.g. Cohen's *d*, Pearson's *r*), indicating how they were calculated

Our web collection on statistics for biologists contains articles on many of the points above.

Software and code

Policy information about availability of computer code

Data collection	No software was used for data collection
Data analysis	Statistics software package R v.3.1.0 and Microsoft Excel 2007 were used for statistical analysis. GraphPad Prism 7.04 was used for the preparation of the scatter plots of the AHLs levels in the samples.

For manuscripts utilizing custom algorithms or software that are central to the research but not yet described in published literature, software must be made available to editors and reviewers. We strongly encourage code deposition in a community repository (e.g. GitHub). See the Nature Research guidelines for submitting code & software for further information.

Data

Policy information about availability of data

All manuscripts must include a data availability statement. This statement should provide the following information, where applicable:

- Accession codes, unique identifiers, or web links for publicly available datasets
- A list of figures that have associated raw data
- A description of any restrictions on data availability

All data generated in method development are provided in Support Information. The source data underlying Figs. 1-5 and all other data are available from the corresponding author on reasonable request.

Field-specific reporting

Please select the one below that is the best fit for your research. If you are not sure, read the appropriate sections before making your selection.

☒ Life sciences ☐ Behavioural & social sciences ☐ Ecological, evolutionary & environmental sciences

For a reference copy of the document with all sections, see nature.com/documents/nr-reporting-summary-flat.pdf

Life sciences study design

All studies must disclose on these points even when the disclosure is negative.

Sample size	n=8
Data exclusions	No data were excluded from the analysis.
Replication	The results are consistent in biological replicates, with 8 animals being analyzed.
Randomization	All serum and tissue samples were collected following in the same protocol. Samples were randomly injected to LC-MS for AHL analysis, and the level of AHL is automatically calculated by a predefined integrating method.
Blinding	Blinding was not relevant to this study because all AHL concentration was determined by software automatically but not manually.

Reporting for specific materials, systems and methods

We require information from authors about some types of materials, experimental systems and methods used in many studies. Here, indicate whether each material, system or method listed is relevant to your study. If you are not sure if a list item applies to your research, read the appropriate section before selecting a response.

Materials & experimental systems

n/a	Involved in the study	
☒	☐	Antibodies
☒	☐	Eukaryotic cell lines
☒	☐	Palaeontology and archaeology
☐	☒	Animals and other organisms
☒	☐	Human research participants
☒	☐	Clinical data
☒	☐	Dual use research of concern

Methods

n/a	Involved in the study	
☒	☐	ChIP-seq
☒	☐	Flow cytometry
☒	☐	MRI-based neuroimaging

Animals and other organisms

Policy information about studies involving animals; ARRIVE guidelines recommended for reporting animal research

Laboratory animals	specific-pathogen free (SPF) C57BL/6 mice
Wild animals	This study didn't involve wild animals
Field-collected samples	The mice were housed under the environmental conditions of 22 °C, 40-70% humidity, and a 12:12 h light/dark cycle and were provided water ad libitum throughout the experiment period. Regular monitoring for health conditions was twice a week. After 7 weeks, serum, urine, liver, cecal content, and feces samples from individual mouse were collected and kept at -80 °C immediately for further analysis. Germ-free mice (~8-week-old) were maintained in UNC germ-free animal facility and samples of germ-free mice were collected and stored as SPF mice.
Ethics oversight	All animal study plan and relevant ethics were approved by the University of North Carolina at Chapel Hill and Massachusetts Institute of Technology Institutional Animal Care and Use Committees.

Note that full information on the approval of the study protocol must also be provided in the manuscript.
