# Supplementary material for: Detection of gut microbiota and pathogen produced N-acyl homoserine in host circulation and tissues
Source: NPJ Biofilms Microbiomes. 2021 Jun 28;7:53. doi: 10.1038/s41522-021-00224-5 (PMC8239043; doi:10.1038/s41522-021-00224-5)
Supplement: Supplementary file 2 — Supplementary Information [file 41522_2021_224_MOESM2_ESM.pdf]

Supplementary Information for

Gut Microbiota Produced N-Acyl Homoserine Lactones and Their Trans-Kingdom Transportation

Jingchuan Xue[†,#], Liang Chi[†,#], Pengcheng Tu[†], Yunjia Lai[†], Chih-Wei Liu[†], Hongyu Ru[†], and Kun Lu[†,*]

[†] Department of Environmental Sciences and Engineering, University of North Carolina at Chapel Hill, Chapel Hill, NC 27599;

* Corresponding author
Kun Lu, PhD
Department of Environmental Sciences and Engineering
University of North Carolina at Chapel Hill, Chapel Hill, NC 27599
Tel.: 919 966 7337
Email: kunlu@unc.edu

#: equal contribution

Supplementary Table 1. List of quorum sensing molecules measured in this study

No.	Trivial_Name	CAS No.	Catalog#	Vendor	Molecular Formula	Exact Mass
1	C4-HSL	202284-85-3	10007898	Cayman	C8H13NO3	171.08954
2	C6-HSL	147852-83-3	10007896	Cayman	C10H17NO3	199.12084
3	C7-HSL	177158-20-2	10011198	Cayman	C11H19NO3	213.13649
4	C8-HSL	147852-84-4	10011199	Cayman	C12H21NO3	227.15214
5	C9-HSL	177158-21-3	16868	Cayman	C13H23NO3	241.16779
6	C10-HSL	177315-87-6	10011201	Cayman	C14H25NO3	255.18343
7	C11-HSL	216596-71-3	16827	Cayman	C15H27NO3	269.19908
8	C12-HSL	137173-46-7	10011203	Cayman	C16H29NO3	283.21473
9	C13-HSL	878627-21-5	13093	Cayman	C17H31NO3	297.23038
10	C14-HSL	202284-87-5	10011200	Cayman	C18H33NO3	311.24603
11	C15-HSL	182359-66-6	13094	Cayman	C19H35NO3	325.26168
12	C16-HSL	87206-01-7	13064	Cayman	C20H37NO3	339.27733
13	C18-HSL	479050-96-9	13209	Cayman	C22H41NO3	367.30863
14	C14-9Z-HSL	1675245-06-3	10012672	Cayman	C18H31NO3	309.23038
15	C16-9Z-HSL	479050-94-7	10012673	Cayman	C20H35NO3	337.26168
16	C18-9Z-HSL	1400974-23-3	10012674	Cayman	C22H39NO3	365.29298
17	3-oxo-C6(L)-HSL	143537-62-6	10011207	Cayman	C10H15NO4	213.100103
18	3-oxo-C8-HSL	147795-39-9	10011206	Cayman	C12H19NO4	241.1314014
19	3-oxo-C10-HSL	147795-40-2	O9014	Sigma-Aldrich	C14H23NO4	269.1626998
20	3-oxo-C12-HSL	168982-69-2	O9139	Sigma-Aldrich	C16H27NO4	297.193998
21	3-oxo-C14-HSL	177158-19-9	13063	Cayman	C18H31NO4	325.225297
22	3-oxo-C16-HSL	925448-37-9	13062	Cayman	C20H35NO4	353.256595
23	3-OH-C8-HSL	192883-14-0	9001150	Cayman	C12H21NO4	243.14705
24	3-OH-C10-HSL	192883-12-8	9001147	Cayman	C14H25NO4	271.178349
25	3-OH-7Z-C14-HSL	273734-65-9	9002939	Cayman	C18H31NO4	325.225297
26	3-oxo-7Z-C14-HSL	482598-46-9	13213	Cayman	C18H29NO4	323.20965
27	3-oxo-C16-11Z-HSL	1269663-80-0	10011238	Cayman	C20H33NO4	351.24095

Supplementary Table 2. MRM transitions of quorum sensing molecules measured in this study.

No.	compound	Q1	Q3	Q3
1	C4-HSL	172.09682	102.05496	71.049144
2	C6-HSL	200.12812	102.05496	99.080444
3	C7-HSL	214.14377	102.05496	113.096094
4	C8-HSL	228.15942	102.05496	127.111744
5	C9-HSL	242.17507	102.05496	141.127394
6	C10-HSL	256.19071	102.05496	155.143034
7	C11-HSL	270.20636	102.05496	169.158684
8	C12-HSL	284.22201	102.05496	183.174334
9	C13-HSL	298.23766	102.05496	197.189984
10	C14-HSL	312.25331	102.05496	211.205634
11	C15-HSL	326.26896	102.05496	225.221284
12	C16-HSL	340.28461	102.05496	239.236934
13	C18-HSL	368.31591	102.05496	267.268234
14	C14-9Z-HSL	310.23766	102.05496	209.189984
15	C16-9Z-HSL	338.26896	102.05496	237.221284
16	C18-9Z-HSL	366.30026	102.05496	265.252584
17	3-oxo-C6(L)-HSL	214.107383	102.05496	113.059707
18	3-oxo-C8-HSL	242.1386814	102.05496	141.0910054
19	3-oxo-C10-HSL	270.1699798	102.05496	169.1223038
20	3-oxo-C12-HSL	298.201278	102.05496	197.153602
21	3-oxo-C14-HSL	326.232577	102.05496	225.184901
22	3-oxo-C16-HSL	354.263875	102.05496	253.216199
23	3-OH-C8-HSL	244.15433	102.05496	143.106654
24	3-OH-C10-HSL	272.185629	102.05496	171.137953
25	3-OH-7Z-C14-HSL	326.232577	102.05496	225.184901
26	3-oxo-7Z-C14-HSL	324.21693	102.05496	223.169254
27	3-oxo-C16-11Z-HSL	352.24823	102.05496	251.24095

Supplementary Table 3. Sensitivity and linearity of quorum sensing molecules.

No.	compound	LLOD	Linearity (Y, nM; X, A_n/A_i)	R^2
1	C4-HSL	1	Y=0.0179259+0.00468605*X	0.9991
2	C6-HSL	0.2	Y=0.00476228+0.0250249*X	0.9994
3	C7-HSL	0.1	Y=0.00194657+0.072757*X	0.9989
4	C8-HSL	0.5	Y=0.0302568+0.0794892*X	0.9993
5	C9-HSL	0.05	Y=-0.000413845+0.168043*X	0.9991
6	C10-HSL	0.01	Y=0.00850033+0.156812*X	0.9993
7	C11-HSL	0.01	Y=0.00737943+0.201798*X	0.9997
8	C12-HSL	0.1	Y=0.00738679+0.0337526*X	0.9990
9	C13-HSL	0.02	Y=-0.00303667+0.16336*X	0.9984
10	C14-HSL	0.05	Y=0.0031114+0.0210947*X	0.9984
11	C15-HSL	0.02	Y=0.00760983+0.0937509*X	0.9967
12	C16-HSL	0.05	Y=0.00765347+0.0718923*X	0.9979
13	C18-HSL	0.05	Y=0.0110782+0.0765107*X	0.9993
14	C14-9Z-HSL	0.02	Y=-0.0033186+0.215736*X	0.9986
15	C16-9Z-HSL	0.05	Y=0.00551776+0.0769918*X	0.9982
16	C18-9Z-HSL	0.05	Y=0.00143497+0.0931235*X	0.9984
17	3-oxo-C6(L)-HSL	0.2	Y=-0.0144746+0.033232*X	0.9825
18	3-oxo-C8-HSL	0.05	Y=-0.00174594+0.122143*X	0.9998
19	3-oxo-C10-HSL	0.02	Y=0.00699162+0.120807*X	0.9998
20	3-oxo-C12-HSL	0.02	Y=0.00118023+0.314625*X	0.9996
21	3-oxo-C14-HSL	0.1	Y=-0.00405702+0.096408*X	0.9990
22	3-oxo-C16-HSL	0.01	Y=0.0260118+0.217612*X	0.9996
23	3-OH-C8-HSL	0.02	Y=-0.00428251+0.210093*X	0.9988
24	3-OH-C10-HSL	0.01	Y=-0.00111204+0.294394*X	0.9993
25	3-OH-7Z-C14-HSL	0.05	Y=0.0526096+0.337221*X	0.9994
26	3-oxo-7Z-C14-HSL	0.05	Y=0.00812783+0.221829*X	0.9997
27	3-oxo-C16-11Z-HSL	0.05	Y=-0.00772647+0.233827*X	0.9985

Supplementary Table 4. Limit of quantification of quorum sensing molecules in different sample types.

No.	compound	LLOQ (serum)	LLOQ (liver)
1	C4-HSL	2	5
2	C6-HSL	0.5	2
3	C7-HSL	0.2	0.5
4	C8-HSL	1	1
5	C9-HSL	0.05	0.1
6	C10-HSL	0.05	0.1
7	C11-HSL	0.05	0.2
8	C12-HSL	0.5	1
9	C13-HSL	0.05	0.2
10	C14-HSL	0.2	0.5
11	C15-HSL	0.1	0.2
12	C16-HSL	0.1	0.1
13	C18-HSL	0.1	0.2
14	C14-9Z-HSL	0.05	0.2
15	C16-9Z-HSL	0.2	0.2
16	C18-9Z-HSL	0.1	0.1
17	3-oxo-C6(L)-HSL	0.5	1
18	3-oxo-C8-HSL	0.1	0.5
19	3-oxo-C10-HSL	0.05	0.5
20	3-oxo-C12-HSL	0.05	0.5
21	3-oxo-C14-HSL	0.2	1
22	3-oxo-C16-HSL	0.02	0.2
23	3-OH-C8-HSL	0.05	0.2
24	3-OH-C10-HSL	0.02	0.2
25	3-OH-7Z-C14-HSL	0.1	0.5
26	3-oxo-7Z-C14-HSL	0.1	1
27	3-oxo-C16-11Z-HSL	0.1	1

Supplementary Table 5. Matrix effects (%) of quorum sensing molecules in different sample types.

No.	compound	serum	liver
1	C4-HSL	109±11	74±3.2
2	C6-HSL	116±2	79±13
3	C7-HSL	104±7	87±16
4	C8-HSL	102±2	88±19
5	C9-HSL	110±6	87±13
6	C10-HSL	101±7	72±18
7	C11-HSL	93±2	74±8.8
8	C12-HSL	98±11	64±26
9	C13-HSL	101±9	80±15
10	C14-HSL	108±7	88±4
11	C15-HSL	96±2	73±10
12	C16-HSL	105±15	88±25
13	C18-HSL	98±3	86±19
14	C14-9Z-HSL	99±6	73±15
15	C16-9Z-HSL	99±14	71±20
16	C18-9Z-HSL	114±7	68±25
17	3-oxo-C6(L)-HSL	115±16	87±10
18	3-oxo-C8-HSL	120±6	68±12
19	3-oxo-C10-HSL	96±9	79±22
20	3-oxo-C12-HSL	107±4	67±25
21	3-oxo-C14-HSL	111±8	68±19
22	3-oxo-C16-HSL	115±7	74±15
23	3-OH-C8-HSL	124±2	84±18
24	3-OH-C10-HSL	101±7	83±8.8
25	3-OH-7Z-C14-HSL	100±6	70±8.6
26	3-oxo-7Z-C14-HSL	109±5	70±8.1
27	3-oxo-C16-11Z-HSL	97±9	51±16

Supplementary Table 6. Accuracy and precision (RSD) of in quantitation of quorum sensing molecules in serum and liver samples.

compound	Serum						Liver					
	MS-L (n=6)			MS-H (n=6)			MS-L (n=6)			MS-H (n=6)		
	recovery %	precision, RSD % (n=6)		recovery %	precision, RSD % (n=6)		recovery %	precision, RSD % (n=6)		recovery %	precision, RSD % (n=6)	
		intra-day	inter-day		intra-day	inter-day		intra-day	inter-day		intra-day	inter-day
C4-HSL	75.6	21.6	23.3	73.9	12.1	9.28	44.9	9.31	12.1	59.9	21.7	26.1
C6-HSL	90.7	10.8	7.43	88.6	5.29	6.03	83.9	9.53	7.49	102	8.59	6.58
C7-HSL	99.4	6.38	16.1	94.1	12.8	1.46	100	11.8	12	112	5.76	7.11
C8-HSL	96.9	10.6	11.7	92.2	11.8	3.23	95.3	11	7.12	99	7.63	7
C9-HSL	96.4	20.2	28.7	85.4	17.1	4.53	109	5.56	6.65	106	8.81	8.37
C10-HSL	100	17.4	27	91.4	16.5	7.42	115	13.6	13.4	116	9.96	10.4
C11-HSL	115	4.33	22.7	114	18.9	17.1	104	3.31	4.04	113	9.91	10
C12-HSL	93	18.8	14.7	111	13.2	6.14	75.2	17.7	16.6	63.2	17.2	16.8
C13-HSL	83.8	10.7	25.3	74.5	17.4	10.6	105	11	9.21	124	11.9	13.1
C14-HSL	70.9	14.2	16	77.9	12.3	8.45	122	17.1	17.3	125	9.06	11.7
C15-HSL	92.9	23.1	27.9	94.8	28.3	8.93	113	10.5	13.6	118	14.6	16.8
C16-HSL	63.5	19.7	27.2	72.6	26.5	11	86.8	10.5	18.4	113	12.4	15.6
C18-HSL	69.5	29.7	26.5	61	22.3	19	52.9	26.4	26.2	129	19.3	29.4
C14-9Z-HSL	82.2	21	20.8	88.2	23.4	13.2	112	11	13.4	103	11	11.3
C16-9Z-HSL	76.3	27	27.5	98.1	21.4	24	92	2.58	11	109	24.5	25.4
C18-9Z-HSL	76.8	24.3	23.3	74.8	16.7	10.1	99.8	14.8	16.7	103	12.8	14.1
3-oxo-C6(L)-HSL	91.1	17.5	16.5	84.1	27.5	9.72	57.5	24	23.6	66.8	24.3	25.4
3-oxo-C8-HSL	100	5.32	19	96.6	5.59	3.63	109	12.4	12.5	100	6.7	7.56
3-oxo-C10-HSL	87.4	11.8	6.53	89.7	14.3	8.37	109	8.94	6.43	90.2	13.3	14.1
3-oxo-C12-HSL	98	13.7	20.5	86.6	20.6	4.39	126	10.7	10.7	115	13.5	13.6
3-oxo-C14-HSL	75	15.1	26.7	80	13.7	13	105	12.3	18.3	86.2	11.5	12.7
3-oxo-C16-HSL	87.6	19.2	16.6	72.4	16.4	6.18	122	12.2	10.7	128	21.1	21.3
3-OH-C8-HSL	99.8	5.32	5.89	88.4	9.08	4.16	106	6.92	7.58	95.3	7.31	7.14
3-OH-C10-HSL	97.6	18.5	26.2	81.9	18.7	4.24	109	5.06	2.25	105	6.43	6.24
3-OH-7Z-C14-HSL	73.2	18.4	28.8	88.5	17.8	13.4	53.6	11.2	10.8	61.5	6.78	4.17
3-oxo-7Z-C14-HSL	78.2	21.7	22.2	86	13.2	7.6	79.6	21.7	19.8	93.5	13.5	13.6
3-oxo-C16-11Z-HSL	87.1	15.2	26	74.1	11.9	10.3	117	11.9	16	128	18	18.7

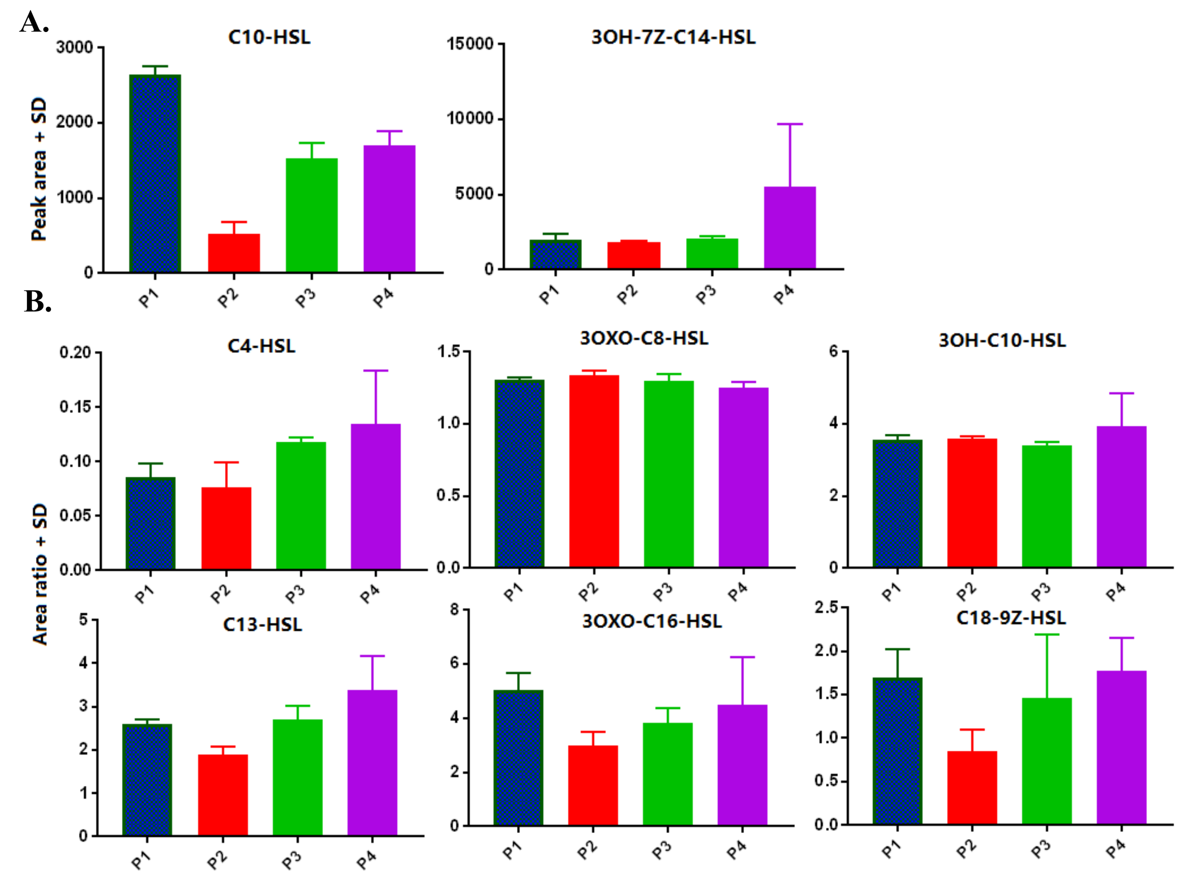

Supplementary Figure 1. Comparison of four serum sample preparation approaches including PPT-SPE (P1), RP-SPE (P2), NP-SPE (P3), and PD-SPE (P4). Fig. A shows the comparison of extraction efficiency of AHLs extracted from serum test sample based on peak area plus standard deviation (SD). Fig. B shows the comparison of extraction efficiency of AHLs spiked into the serum test sample based on area ratio plus standard deviation (SD).

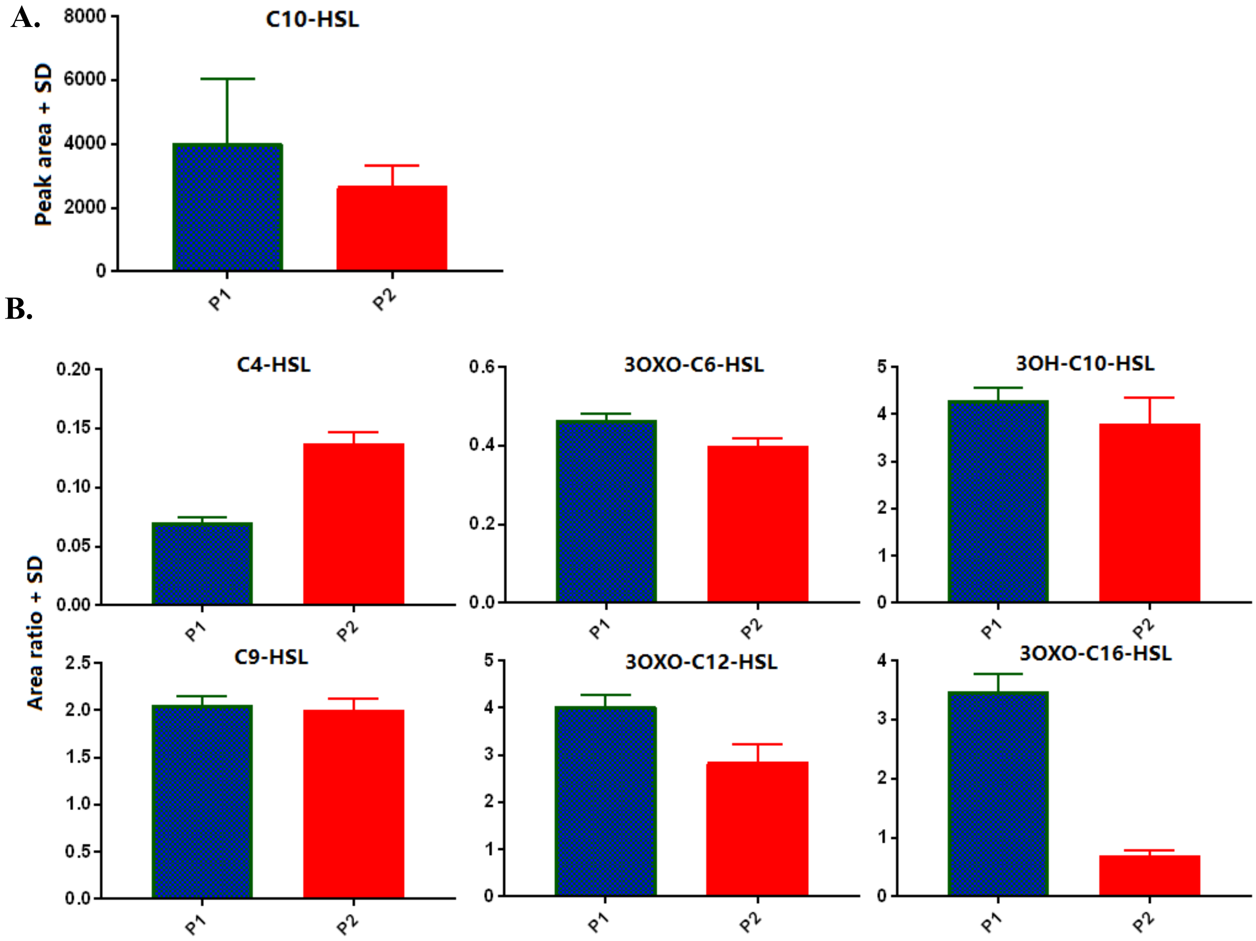

Supplementary Figure 2. Comparison of two liver sample preparation approaches including RP-SPE (P1) and NP-SPE (P2). Fig. A shows the comparison of extraction efficiency of AHLs extracted from liver test sample based on peak area plus standard deviation (SD). Fig. B shows the comparison of extraction efficiency of AHLs spiked into the liver test sample based on area ratio plus standard deviation (SD).

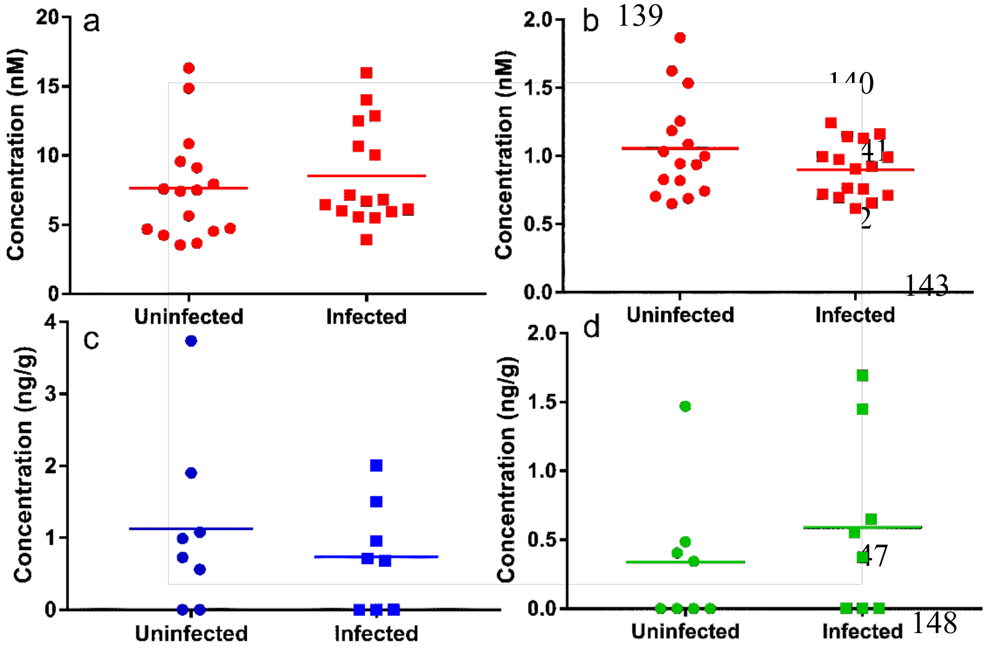

Supplementary Figure 3. Comparison of other AHLs detected in both uninfected and infected samples: (a) C14-HSL in serum; (b) 3OH-C8-HSL in serum; (c) C14-HSL in cecal matter; (d) C14-HSL in liver. The unit is nM in serum samples (n=16 each) and ng/g in liver and cecal matter samples (n=8 each).

References

1 Catharine A. Ortori, N. H., Miguel Cámara, Paul Williams, and David A. Barrett. (Springer, 2014).

2 Gould, T. A., Herman, J., Krank, J., Murphy, R. C. & Churchill, M. E. Specificity of acyl-homoserine lactone synthases examined by mass spectrometry. *J Bacteriol* **188**, 773-783, doi:10.1128/JB.188.2.773-783.2006 (2006).

3 Zhang, W. & Li, C. Exploiting Quorum Sensing Interfering Strategies in Gram-Negative Bacteria for the Enhancement of Environmental Applications. *Front Microbiol* **6**, 1535, doi:10.3389/fmicb.2015.01535 (2015).

4 Ortori, C. A. *et al.* Simultaneous quantitative profiling of N-acyl-l-homoserine lactone and 2-alkyl-4(1H)-quinolone families of quorum-sensing signaling molecules using LC-MS/MS. *Anal Bioanal Chem* **399**, 839-850, doi:10.1007/s00216-010-4341-0 (2011).
